# Side Effects of Novel Anticancer Drugs on the Posterior Segment of the Eye: A Review of the Literature

**DOI:** 10.3390/jpm14121160

**Published:** 2024-12-19

**Authors:** Filippo Lixi, Giuseppe Giannaccare, Giulio Salerno, Vincenzo Gagliardi, Alfonso Pellegrino, Livio Vitiello

**Affiliations:** 1 Eye Clinic, Department of Surgical Sciences, University of Cagliari, 09124 Cagliari, CA, Italy; f.lixi0106@gmail.com; 2 Eye Unit, “Luigi Curto” Hospital, Azienda Sanitaria Locale Salerno, 84035 Polla, SA, Italy; giuliosalerno@hotmail.it (G.S.); v.gagliardi@aslsalerno.it (V.G.); al.pellegrino@aslsalerno.it (A.P.); livio.vitiello@gmail.com (L.V.)

**Keywords:** anticancer drugs, eye, posterior segment, retina, side effects

## Abstract

Currently, common treatment approaches for neoplastic diseases include surgery, radiation, and/or anticancer drugs (chemotherapy, hormone medications, and targeted therapies). In particular, anticancer medicines destroy cancerous cells by blocking certain pathways that aid in the disease’s initiation and progression. These pharmaceutical drugs’ capacity to inhibit malignant cells has made them indispensable in the treatment of neoplastic disorders. Nonetheless, considering their cyto- and neurotoxicity, as well as their inflammatory responses, these medications may also have unfavorable systemic and ocular side effects. In fact, it is well known that ocular posterior segment side effects, including retinal and vascular complications, have a negative influence on the patient’s eyesight and quality of life. However, the underlying mechanisms contributing to the development of these side effects remain incompletely recognized, especially in the case of newly available anticancer drugs. The purpose of this literature review is to analyze the possible side effects of new anticancer drugs on the posterior segment of the eye, trying to better understand the involved pharmacological mechanisms and offer helpful guidance on their appropriate management.

## 1. Introduction

Cancer is characterized by the body’s cells proliferating and dividing abnormally and uncontrollably. According to predictions, by 2030, there will likely be 23.6 million cases of cancer worldwide, putting it among the main reasons for death. Notwithstanding this number, efforts to create novel medications, early detection, and cancer screening systems have improved overall cancer survival over the past few decades [1]. In this regard, new drugs are continuously being developed with the aim of effectively stopping the progression of the disease or preventing further detrimental effects. In fact, novel and innovative anticancer treatments have been developed to target one or more facets of the pathophysiology of the neoplastic disease. The most recent anticancer medications include immune checkpoint inhibitors in cancer immunotherapy, epidermal or fibroblastic growth factor receptor inhibitors, and a constantly expanding range of monoclonal antibodies that are directed against cancerous conditions.

Currently, the potential side effects of anticancer drugs on the ocular surface are well known and well documented [2].

However, these drugs may also cause vision-threatening adverse effects affecting the posterior segment of the eye [3,4]. In particular, the retina plays a crucial role in converting light into neural signals, enabling vision. Its complex structure includes several neuronal layers, photoreceptors, and a dense vascular network crucial for metabolic support. As a result, the posterior segment of the eye, and particularly the retina and the choroid, are highly vulnerable to systemic changes and drug-induced toxicities, given their intricate physiology and dependence on a delicate balance of metabolic processes [3,4]. For this reason, recognizing and promptly managing posterior segment adverse reactions could be crucial for the patients’ eyesight and quality of life.

The purpose of this review is to present a thorough overview of the potential side effects of recently introduced anticancer drugs on the posterior segment of the eye, to gain a deeper comprehension of the pharmacological processes that underlie the development of these adverse events, and to provide valuable guidance on how to effectively manage them.

## 2. Materials and Methods

We performed a search on the PubMed medical database. The words “ocular posterior segment”, “vitreous”, “choroid”, “uvea”, “optic nerve”, and “retina” were used in conjunction with a number of other “text words” pertaining to anticancer drugs (such as “epidermal growth factor receptor inhibitors”, “fibroblast growth factor receptor inhibitors”, “human epidermal growth factor 2 inhibitors”, “immune checkpoint inhibitors”, “mitogen-activated protein kinase inhibitors”, “proto-oncogene BRAF inhibitors”, “aromatase inhibitors”, “selective estrogen receptor modulators”, “Bruton tyrosine kinase inhibitors”, “breakpoint cluster region-Abelson oncogene inhibitors”, “vascular endothelial growth factor receptor inhibitors”, “proteasome inhibitors”, “anaplastic lymphoma kinase inhibitors”, “FMS-like tyrosine kinase 3 inhibitors”, “novel anticancer drugs”) AND possible side effects on the posterior segment of the eye (such as “floaters”, “optic neuropathy”, “retinopathy”, “choroidopathy”).

All terms were selected based on a review of the current literature and relevant bibliographies. The search was completed in November 2024, covering publications from January 1990 onwards. No language restrictions were applied during the search process; however, only papers written in English were analyzed. Additionally, manual searches from the original results were also carried out to find more pertinent bibliographic sources.

## 3. Results

### 3.1. Epidermal Growth Factor Receptor Inhibitors

The epidermal growth factor receptor (EGFR) is a tyrosine kinase crucial for angiogenesis, apoptosis, cell division, and the spread of metastases [5].

Furthermore, additional signaling pathways, including RAS/RAF/MEK, STAT, and PI3K/AKT/mTOR, all closely associated with carcinogenesis, may be activated by EGFR. Consequently, many malignancies, including those of the lungs, kidneys, prostate, breast, pancreas, head, colon, neck, brain, and ovaries, have been connected to the overexpression of EGFR [5].

Given the crucial role that EGFR plays in the development of cancer, inhibitors were created. The first-generation drugs were erlotinib and gefitinib, while the second-generation ones were osimertinib, cetuximab, afatinib, brigatinib, dacomitinib, panitumumab, and necitumumab [5].

The main ophthalmic side effects of EGFR inhibitors are related to the cornea, considering that EGFR is essential for the healing of corneal wounds [2]. Nonetheless, some EGFR inhibitors were also found to develop retinal complications. In fact, gefitinib and brigatinib have been shown to cause macular edema in 0.5% and 0.9% of patients, respectively [6,7].

In addition, floaters were found in 0.5% of patients receiving gefitinib [6].

Although these adverse effects have been described, drug discontinuation was not needed, and no valid pathophysiological mechanism has been hypothesized.

### 3.2. Fibroblast Growth Factor Receptor Inhibitors

Fibroblast growth factor receptors (FGFRs) are tyrosine kinases activated by several fibroblast growth factor (FGF) ligands, which are extensively expressed in all adult tissues [8]. FGFRs have been connected to carcinogenesis, particularly for ovarian cancer, hepatocellular carcinoma, urothelial carcinoma, and lung adenocarcinoma [9].

Erdafitinib and ponatinib are the two primary FGFR inhibitors. Erdafitinib is an FGFR inhibitor specifically approved for the cure of metastatic urothelial carcinoma [10], but it is also being studied for esophageal malignancy, lymphoma, prostate cancer, hepatocarcinoma, non-small cell lung cancer, and cholangiocarcinoma [9].

While there were no ocular side effects observed with ponatinib, 6% of patients on erdafitinib required permanent discontinuation secondary to retinal disorders [10], such as retinal detachment, retinal edema, and chorioretinopathy. Moreover, another 23% of patients on erdafitinib required dose reductions specifically due to eye disorders [10].

According to Loriot and colleagues, 21% of patients developed central serous retinopathy, necessitating stopping medication [11]. In 0.5% and 1% of patients, respectively, retinopathy and chorioretinopathy were also documented; medication holidays or discontinuations were used to manage both adverse effects [11]. Drug cessation was also used to treat the 1% of individuals who had bilateral exudative retinal detachment and edema [11].

Retinal pigment epithelium (RPE) detachments were also reported by Loriot et al. in 0.9% and Bahleda et al. in 0.5% of cases, and in both instances, it was necessary to stop the medication [11,12].

As with EGFR inhibitors, it was not possible to hypothesize any pathophysiological mechanisms underlying these rare retinal complications.

### 3.3. Human Epidermal Growth Factor 2 Inhibitors

About 25% of patients with breast cancer had overexpression or detection of a tumor-associated antigen known as human epidermal growth factor receptor 2 (HER2) [13]. Since their initial development, HER2 inhibitors have increased median overall survival by 57 months [13]. The initial HER2 inhibitors, trastuzumab and pertuzumab, have been supplemented with novel HER2 inhibitors, including lepatinib, neratinib, and tucatinib.

According to the Food and Drug Administration (FDA) and the published scientific literature, only trastuzumab has been linked to retinal side effects, whereas pertuzumab, lapatinib, neratinib, and tucatinib have not been linked to any ocular side effects.

In fact, a case report of bilateral macular ischemia and edema that manifested three months after trastuzumab introduction for breast cancer was reported by Saleh et al. [14].

Furthermore, trastuzumab is an antiangiogenic agent inhibiting vascular endothelial growth factor (VEGF), and ischemic maculopathy has also been reported with the concomitant use of intravitreal bevacizumab [15]. For this reason, macular ischemia linked to the use of trastuzumab could be related to its antiangiogenic activity. Therefore, particular attention should be paid in the case of combined use of trastuzumab and intravitreal therapy with anti-VEGF drugs.

Finally, other potential side effects of trastuzumab include papilledema, retinal hemorrhage, retinal artery occlusion, and retinal vein occlusion [16].

### 3.4. Immune Checkpoint Inhibitors

In recent years, immunotherapy has become an ultramodern treatment for a number of cancers, and immune checkpoint inhibitors are now being utilized for the treatment of lung and pancreatic cancers, as well as lymphoma and melanoma.

The purpose of immune checkpoint inhibitors is to increase T lymphocytes’ immune response against tumor cells by blocking immunological checkpoint molecules like programmed cell death protein 1 (PD-1), cytotoxic T-lymphocyte-associated protein 4 (CTLA-4), and programmed death ligand 1 (PD-L1).

The first immune checkpoint inhibitor, ipilimumab, was approved by FDA in 2011 for metastatic or incurable cutaneous melanoma [3]. Since then, the following drugs have been approved: atezolizumab, durvalumab, nivolumab, pembrolizumab, and cemiplimab [17,18,19,20,21].

The list of these new medications’ indications and off-label uses has grown over time due to their demonstrated survival benefit in a variety of cancers, such as Hodgkin lymphoma, colorectal cancer, gastric cancer, urothelial carcinoma, metastatic non-small cell lung cancer, hepatocarcinoma, and head and neck squamous cell carcinoma [22]. Unfortunately, their extensive and nonspecific immunological activity may result in undesired adverse effects on the systemic immune system. According to reports, ocular immune-related adverse events are less prevalent than systemic immune-related adverse events, occurring in around 1–2.8% of patients taking immune checkpoint inhibitors [23].

Inflammation related to immune checkpoints inhibitors, which affects around 1% of patients, can involve the orbit, extraocular muscles, cranial nerves, ocular surface, uvea, retina, and optic nerve [24,25,26,27]. Concerning the posterior segment of the eye, Vogt–Koyanagi–Harada (VKH)-like syndrome [28,29], panuveitis [30], and posterior uveitis [31] are all possible. Uveitis can be linked to severe hypotony [32] or papillitis [33]. Neurosensory detachment without choroidopathy [34], multifocal neurosensory detachments with choroidal thickening without inflammation [35], choroidal neovascular membranes [36], and immunological retinopathy [37] have all been previously reported. Topical, periocular, or systemic corticosteroids are used to reduce inflammation, and intravitreal corticosteroids are used to treat any related macular edema, in addition to drug discontinuation in severe cases that are not responding to treatment.

With an estimated prevalence of 0.43%, optic neuritis has also been linked to immune checkpoint inhibitors [38]. Intravenous methylprednisolone and high-dose oral prednisone are the usual treatments for optic neuritis associated with these drugs. In some cases, intravenous immunoglobulin and plasma exchange therapy are also necessary. Most cases with optic neuritis necessitated stopping treatment, according to Yu et al. [38]. Sometimes, immune checkpoint inhibitors have also been linked to giant cell arteritis, starting as retinal artery occlusion and paracentral acute middle maculopathy [39,40,41]. High-dose oral prednisone was used to treat each of these patients but, considering the risk of irreversible eyesight loss, the treatment was also stopped.

There are unique special mechanisms against inflammation and infection inside the eye, but it is unclear how they interact with checkpoint immune inhibitors. Ocular immune privilege encompasses both anatomical predisposition, such as the absence of efferent lymphatics and the blood retinal barrier, and immunological mechanisms, such as the overexpression of Fas ligand and tumor growth factor beta. This prevents inflammation by causing immune cell death and converting T cells to regulatory T cells [42]. In addition, RPE cells that interact with CTLA-4 and PD-1 may express CD86 and PD-L1, respectively, thus preventing inflammation caused by T cells [43]. It is thought that immune checkpoint inhibitors affect ocular tissues via interference with the ocular immune privilege.

### 3.5. Mitogen-Activated Protein Kinase Inhibitors

Mitogen-activated protein kinase (MEK) is an enzyme within the mitogen-activated protein kinase (MAPK) pathway, which controls different physiological processes, including gene expression, cell cycle control, and proliferation. MEK and its pathway may overactivate in various cancers [44]. Precisely, MEK inhibitors have been utilized for the treatment of BRAF-mutated melanoma, KRAS/BRAF-mutated colorectal cancer, hepatocellular carcinoma, low-grade serous ovarian cancer, biliary cancers, and metastatic non-small cell lung cancers. Specifically, these drugs act by blocking MEK and disrupting its intracellular signaling cascade. Currently, trametinib, cobimetinib, selumetinib, and binimetinib are the four MEK inhibitors approved by the FDA for clinical use [45].

Several ophthalmic side effects involving either the anterior or the posterior segment of the eye have been documented [2,44].

A recent metanalysis considering 17 randomized controlled trials showed that the use of MEK inhibitors was significantly associated with an increased risk of different retinal side effects [46]. In fact, the MAPK signaling pathway, including MEK, is crucial for maintaining RPE integrity by offering protection against stressors like oxidative stress, light-induced damage, and inflammation. On cultured human RPE cells, MEK inhibition has been shown to cause acute RPE toxicity, leading to increased permeability and disruption of the blood–retina barrier. Curiously, the choroid seems not to be involved [3,45,47].

MEK inhibitor-associated retinopathy (MEKAR) is an umbrella term specifically used to include various entities that have been previously used to describe retinal adverse effects linked to MEK inhibitors, including serous retinopathy (SR), central serous retinopathy (CSR), serous retinal detachment (SRD), macular edema, visual disturbance, chorioretinopathy, and blurred vision [3,45,48].

Currently, MEKAR is considered as a dose- and time- dependent retinal side-effect, which typically presents acutely within the first week following the start of the treatment. It is characterized by single or multiple SRDs, which may progress to the formation of intraretinal fluid associated with the disruption of the outer retinal layers on optical coherence tomography (OCT). Moreover, this condition is often bilateral and frequently symmetrical [3,45].

The wide variability in the incidence of MEKAR may be attributed to differences in the condition’s description, diagnostic criteria, and reporting practices, as well as the frequency of ophthalmic evaluations during clinical trials, especially in asymptomatic patients. However, in a prospective study, its prevalence was observed to range from 90% to 100%; thus, it was considered as a class effect [3,45,49].

Considering that MEKAR is generally asymptomatic, benign, and self-limiting, life-prolonging treatments are typically continued. Conversely, for symptomatic cases characterized by intermittent blurry vision, dark spots in the visual field, and metamorphopsia, dose reductions are usually applied, leading to resolution in most patients [3,49,50,51].

Retinal vein occlusion (RVO) is a serious side effect of MEK inhibitors reported after the use of mirdametinib, trametinib, RO4987655, refametinib, and pimasertib [45,52,53]. Unlike MEKAR, these vascular events occurred generally months after the initiation of therapy. According to FDA guidelines, in cases of RVO, treatment for macular edema with anti-VEGF injections was applied and a discontinuation of MEK inhibitor was required [54,55,56,57].

In addition, MEK inhibitors have also been linked to posterior uveitis and panuveitis, including Vogt–Koyanagi–Harada-like syndrome [58,59,60,61].

Several treatments have been explored, including topical, periocular, or systemic corticosteroids to manage inflammation, as well as cycloplegic drugs and medications to lower intraocular pressure [58,59,60,61]. If there is no response after specific ocular treatment, therapy may be stopped for up to 6 weeks. Subsequently, if improvement occurs, therapy is then resumed; otherwise, it is permanently discontinued [54,55,56,57].

Therefore, effective communication between oncologists and ophthalmologists is essential to ensure patient safety during MEK inhibitor therapy.

### 3.6. BRAF Inhibitors

In addition to MEK, BRAF is another kinase that plays a key role in the MAPK signaling pathway, and its mutations are implicated in the development of several cancers [44]. Currently, three BRAF inhibitors—vemurafenib, dabrafenib, and encorafenib—are approved by the FDA for the treatment of cancers with BRAF mutations, such as unresectable or metastatic cutaneous melanoma, metastatic non-small cell lung cancer, metastatic anaplastic thyroid cancer, and metastatic colorectal cancer [62,63,64].

Severe cases of uveitis, such as intermediate uveitis, panuveitis, Vogt–Koyanagi–Harada-like syndrome, ischemic retinal vasculitis, and multifocal choroiditis complicated by choroidal neovascularization, have been reported, and these required the discontinuation of BRAF inhibitor therapy, along with specific ocular treatments [65,66,67,68]. Similarly, central RVO cases after vemurafenib use also needed drug discontinuation [69,70].

In addition, dabrafenib and encorafenib were associated with the development of posterior segment complications. In particular, in combination with MEK inhibitors, cases of panuveitis, Vogt–Koyanagi–Harada-like syndrome, retinal vasculitis and MEKAR were recorded. All these conditions, except for MEKAR, necessitated drug discontinuation [49,58,60,71].

The pathogenesis of ocular side effects in patients treated with BRAF inhibitors remains unclear. However, considering that ERK activation in the MAPK pathway promotes both tumor growth and T cell proliferation, and that anti-CTLA4 agents can cause uveitis as a rare side effect, it has been suggested that BRAF inhibitors may induce uveitis through a similar mechanism by activating ERK in certain patients [70].

### 3.7. Aromatase Inhibitors

Aromatase inhibitors are another class of anticancer drugs that work by regulating female hormone levels. They are able to reduce the production of estrogen in peripheral tissues by aromatase enzyme and represent an effective treatment option for hormone-receptor-positive breast cancer in postmenopausal women. Anastrozole, letrozole, and exemestane are the three FDA-approved aromatase inhibitors [72,73,74,75].

Considering the ocular posterior segment, anastrozole and letrozole have been associated with an increased prevalence of retinal hemorrhages, with incidences of 11.4% and 0.05%, respectively [76,77]. The causal mechanism behind this side effect is considered multifactorial and remains incompletely understood. It may result from a systemic cardiovascular effect due to estrogen depletion, leading to reduced blood vessel integrity and thromboembolic predisposition, or from an increased incidence of vitreoretinal traction events, which are common in postmenopausal women with estrogen depletion [76].

Due to its prothrombotic effect, anastrozole has also been linked to a case of hemi-central retinal artery occlusion in a 53-year-old woman with hypertension and diabetes, leading to the drug’s withdrawal [78].

Moreover, changes in vascular permeability secondary to estrogen-level variations were considered as possible causative mechanisms of macular and optic disk edema that were documented after anastrozole administration [79,80,81].

Additionally, a case of crystalline retinopathy was also reported. It is characterized by small, white, refractile crystals in the inner retina, which are characteristically irreversible. These deposits are often asymptomatic, but in symptomatic cases, management may involve dose reduction, or the temporary or permanent discontinuation of the drug. In this case, the patient was closely monitored, with no changes in vision at the 6-month follow-up [82].

Macular edema associated with letrozole administration has also been reported. In fact, in a 72-year-old woman who had been on letrozole therapy for three years, discontinuing the medication and administering ranibizumab led to an improvement in both macular edema and visual acuity [83].

Furthermore, exemestane has been associated with posterior segment collateral effects. Specifically, a case of macular hole was described in a 59-year-old myopic woman, and it was effectively managed with vitrectomy, while a 62-year-old myopic woman discontinued the drug after a retinal detachment in her left eye [84].

### 3.8. Selective Estrogen Receptor Modulator

Female hormones, especially progesterone and estrogen, are primarily responsible for the differentiation and proliferation of breast tissue. Notably, around 70% of cases of breast cancer exhibit up-regulation of the estrogen receptor, highlighting the critical role that endocrine therapy plays in treating breast cancer [85].

Tamoxifen, raloxifene, and toremifene are known as selective estrogen receptor modulators (SERMs). These drugs play a crucial role in breast cancer treatment by acting as tissue-specific agonists or antagonists in their interactions with estrogen receptors [2].

Different studies highlighted the association between SERMs and retinal pathologies. In fact, retinal involvement is generally bilateral and may be accompanied by macular edema [86,87,88]. Its prevalence is estimated to range between 0.9% and 12% [89].

A hallmark of tamoxifen toxicity is crystalline retinopathy [3,87,88].

Furthermore, tamoxifen therapy has also been linked to an increased risk of developing macular holes. It remains unclear whether this side effect is related to estrogenic effects or whether it is independent of hormone levels. The proposed mechanism behind tamoxifen-associated macular hole formation involves toxic damage leading to axonal degeneration, which affects Müller cell function. This impairment might result in the formation of intraretinal foveolar cysts, thereby increasing the risk of macular hole development. Currently, vitrectomy remains the preferred approach [90,91].

Consistent with the thromboembolic risk associated with SERMs, RVO cases related to tamoxifen and toremifene, which required cessation of therapy, have been reported [77,92,93].

Furthermore, cases of optic neuropathy associated with tamoxifen treatment were also described [94,95,96]. Stopping the medication has been shown to improve visual acuity in two cases [95,96].

### 3.9. Bruton’s Tyrosine Kinase Inhibitors

Bruton’s tyrosine kinase (BTK), encoded by the BTK gene, is essential for the development, proliferation, and differentiation of B-cells. BTK inhibitors offer an alternative to chemotherapy-based treatments for B-cell cancers or chronic graft-versus-host disease.

Currently, the FDA-approved BTK inhibitors include ibrutinib, acalabrutinib, and zanubrutinib [97].

In one oncology trial, ibrutinib ocular toxicity was the second most common among anticancer agents. Concerning posterior pole side effects, one patient, suffering from hypertensive retinopathy and hematologic malignancies, presented with branch retinal artery occlusion after ibrutinib therapy and required discontinuation of the drug [98].

Macular edema was also associated with ibrutinib treatment, leading to drug withdrawal and the administration of intravitreal steroids [99,100,101].

The understanding of the mechanism of action of ibrutinib in this context remains speculative. Considering that some tyrosine kinase inhibitors cause uveitis through dysregulation of tight junctions in uveal endothelial cells, a similar mechanism may be proposed for the effect of ibrutinib on the retinal vessels. This would increase vascular permeability, resulting in macular edema [99].

Conversely, no posterior segment side effects have been reported for acalabrutinib or zanubrutinib.

### 3.10. Breakpoint Cluster Region–Abelson Oncogene Inhibitors

The breakpoint cluster region–Abelson oncogene (BCR-ABL), also known as the Philadelphia chromosome, encodes a protein tyrosine kinase that controls cell proliferation, differentiation, and apoptosis. The treatment of chronic myeloid leukemia was revolutionized by imatinib, the first tyrosine kinase inhibitor, which was approved in 2001. Subsequently, the development of second- and third-generation BCR-ABL inhibitors, such as nilotinib, dasatinib, bosutinib, and ponatinib, expanded options for chronic myeloid leukemia management [102].

Among BCR-ABL inhibitors, imatinib has been linked to the greatest incidence of ocular adverse events, with some cases necessitating drug discontinuation [3]. Three cases of macular edema linked to imatinib required treatment with oral prednisone and cessation of the medication. It is worth noting that the understanding of the mechanism behind imatinib-induced macular edema remains speculative. In fact, while imatinib selectively inhibits BCR-ABL tyrosine kinase, it also affects other kinases, including platelet-derived growth factor receptor (PDGFR) and KIT. Since PDGFRs are present in the retina and their down-regulation is linked to edema, imatinib may contribute to this condition in some patients through its inhibitory effects [103,104,105].

Notably, cases of optic nerve edema and SRD have also been reported, which may be attributed to imatinib’s fluid retention activity [106,107,108].

In addition, following imatinib exposure, a potential demyelinating effect may develop. Indeed, some cases of optic neuritis have occurred, and they were managed with drug discontinuation and oral corticosteroids [109,110,111].

Additionally, imatinib has been linked to ischemic maculopathy and retinal hemorrhages, conditions that have typically required dose reduction or treatment cessation [112,113,114].

Furthermore, dasatinib has demonstrated neurotoxic effects on the optic nerve, with two reported cases of optic neuropathy following its use. Both necessitated corticosteroid treatment and drug discontinuation [115,116].

A probable case of ponatinib-induced papilledema was also reported by Moon and colleagues. A 48-year-old patient with acute lymphoblastic leukemia developed bilateral optic disk edema after the initiation of ponatinib therapy. Following treatment with acetazolamide at a dosage of 500 mg twice daily, the edema resolved, and visual recovery was achieved [117].

### 3.11. Vascular Endothelial Growth Factor Inhibitors

Vascular endothelial growth factor (VEGF) is a key pro-angiogenic factor that contributes to the development of both physiological and pathological vascular networks, playing a pivotal role in tumor progression, metastasis, and ocular neovascular disorders. To counteract the activation of pathological pathways in various neoplastic diseases, a range of VEGF and VEGF receptor (VEGFR) inhibitors have been created, such as sorafenib, sunitinib, vandetanib, lenvatinib, axitinib, bevacizumab, ramucirumab, cabozantinib, and pazopanib [118].

Pazopanib is a multikinase inhibitor, mainly used to treat renal cell carcinoma. It was shown to determine different side effects involving the posterior segment of the eye, including retinal detachment or tears, delayed retinal healing after a failed laser retinopexy, vitreous or retinal bleeding, optic nerve disease (papilledema or ischemic optic neuropathy), and retinal artery or vein occlusions. However, the mechanism by which pazopanib may cause collateral effects is not clear [119].

Axitinib, an orally administered VEGFR inhibitor, used as second-line treatment for metastatic renal cell carcinoma, has been linked to two cases of impaired retinal circulation, characterized by multiple cotton-wool spots on fundus examination and significant delays in retinal vessel filling observed on fluorescein angiography. Both cases required discontinuation of the drug [120,121]. Moreover, it is likely that axitinib increases the risk of arterial thromboembolic events due to its potent inhibition of the VEGF signaling cascade [120].

Bevacizumab, a VEGF inhibitor approved for oncological use in metastatic colorectal cancer, non-small cell lung cancer, glioblastoma, and cervical cancer, has been associated to optic neuropathy. One possible mechanism may involve arterial thrombosis or optic nerve ischemia [122].

According to the FDA, cabozantinib has been associated with raised thrombotic events in 6% of patients and should be immediately stopped in case of thrombotic events [123].

No posterior segment side effects have been documented for the other VEGF and VEGFR inhibitors.

### 3.12. Proteasome Inhibitors

The ubiquitin proteasome system is an intracellular selective mechanism that is essential for breaking down unfolded or damaged proteins. Numerous biological functions, such as the cell cycle and apoptosis, depend on it.

Proteasome inhibitors exert anti-cancer effects by deactivating pro-growth proteins and inhibiting cell proliferation. The FDA-approved proteasome inhibitors are bortezomib, ixazomib, and carfilzomib [124].

Bortezomib, the first FDA-approved proteasome inhibitor, is used to treat hematologic malignancies such as mantle cell lymphoma and multiple myeloma. Regarding its toxicity profile on the posterior pole of the eye, two cases of optic atrophy needing instantaneous drug suspension have been reported [125]. Bortezomib is thought to inhibit protein degradation by blocking proteasome activity, resulting in the toxic accumulation of proteins. This buildup can be harmful to cell types with high protein turnover rates, such as retinal ganglion cells [125].

In addition, in a phase 1/1B trial, bortezomib, in association with an inhibitor of heat shock protein 90 (HSP90), caused night blindness and photopsia in two patients affected by multiple myeloma. However, these collateral effects were probably secondary to HSP90 inhibitor-induced photoreceptor degeneration [126].

No further side effects involving the posterior segment of the eye were recorded with ixazomib or carfilzomib.

### 3.13. Anaplastic Lymphoma Kinase Inhibitors

Anaplastic lymphoma kinase (ALK) is a receptor tyrosine kinase identified in cells of anaplastic large-cell lymphoma. Mutations in the ALK gene can result in its oncogenic activation, with the resulting cancers classified as ALK-positive. ALK inhibitors have shown substantial efficacy in treating ALK-positive cancers, particularly for non-small cell lung cancers.

The first ALK inhibitor to receive FDA approval was crizotinib, followed by ceritinib, alectinib, lorlatinib, and entrectinib [127].

Patients on crizotinib frequently experienced vision disorders, including flashes, a post-flashbulb effect, and flipped dark–light perception of high-contrast images at the edges of their visual field, with an incidence rate up to 64%. These visual disorders were generally benign, decreased over time, and did not necessitate therapy [128,129].

In a study of 31 patients on crizotinib, 30.3% experienced a loss of two or more lines in best-corrected visual acuity, and 21.2% showed increased macular thickness on OCT. These side effects did not necessitate permanent discontinuation of the treatment [130].

Similarly, a case of bilateral retinal hemorrhages was reported by Parkish et al. after crizotinib use. The patient was only observed, and the drug was not discontinued [131].

Conversely, for a patient under crizotinib treatment who developed optic neuropathy, temporary discontinuation of the medication was required. However, the ophthalmic symptoms did not resolve upon withdrawal and worsened when treatment was restarted [132].

The next-generation ALK inhibitors exhibit ophthalmic side effect profiles comparable to those of crizotinib, but with lower incidence rates of visual disorders. Patients receiving ceritinib reported vision disturbances with an incidence rate of 9% [133].

The FDA reported that alectinib has a 10% incidence rate for vision disorders, while another study found that 1–2% of patients experienced vision impairment or blurriness [134,135].

Furthermore, the FDA declared that 15% of patients treated with lorlatinib experienced benign vision disorders, which did not require any treatment or changes in therapy [136].

Finally, according to the FDA, entrectinib was shown to have 1.1% and 21% incidence rates of vitreous floaters and vision changes, respectively [137].

### 3.14. Fms-like Tyrosine Kinase 3 Inhibitors

Fms-like tyrosine kinase 3 (FLT3) is part of the class III receptor tyrosine kinase family and regulates the proliferation of hematopoietic cells and lymphocytes. The high frequency of FLT3 mutations across various tumors has driven the development of several targeted FLT3 inhibitors. These inhibitors are classified into first-generation agents with broad spectra and high side effect profiles, including sunitinib, sorafenib, midostaurin, ponatinib, and second-generation agents with greater specificity and reduced toxicity such as quizartinib, crenolanib, regorafenib, and gilteritinib [138].

Sorafenib and sunitinib are multikinase inhibitors targeting RAF-1, VEGF, c-KIT, PDGFR, ERK, and FLT3. In particular, sorafenib has been associated with several cases of retinal arterial and venous occlusions [119]. Li et al. and Szczepanik and Kecik documented two cases of central RVO linked to sorafenib. In one case, the drug was interrupted, and mecobalamine was administered, while in the other case, sorafenib was continued, and the patient received enoxaparin and laser photocoagulation [139,140].

Some reports also described an association between FLT3 inhibitors and retinal tear or detachment [141,142].

Similarly, Fraunfelder and colleagues reported nine and 24 cases of retinal detachment or tear that necessitated sorafenib and sunitinib withdrawal, respectively [119]. Moreover, in the same case series of retinal/vitreous bleeding, macular edema and optic nerve disorders were also described after administration of these two drugs [119].

Bilateral papilledema and optic neuritis have also been reported in patients undergoing sunitinib therapy, requiring drug discontinuation and treatment with systemic methylprednisolone for resolution of these conditions [143,144].

Gilteritinib, another FLT3 inhibitor approved for the treatment of refractory acute myeloid leukemia, has been associated with retinal hemorrhages, which occurred in 7.72% of patients [145].

The possible mechanism for retinal and choroidal complications associated with FLT3 inhibitors involves their extensive inhibition of tyrosine kinase receptors, endothelial growth factors, platelet-derived growth factors, and stem cell factors, which are all implicated in posterior segment vascular and proliferative processes. Additionally, drug-induced changes in vascular permeability, along with possible microemboli formation or microvascular events, may contribute to the observed retinal side effects [119,141].

No ocular side effects have been reported for any of the other FLT3 inhibitors.

### 3.15. Mammalian Target of Rapamycin Inhibitors

The mammalian target of rapamycin (mTOR) plays a key role in the regulation of apoptosis, autophagy, and cell proliferation [146]. For this reason, mTOR inhibitors have being investigated as potential cancer treatments. The FDA has authorized everolimus for the treatment of renal cell carcinoma, progressive neuroendocrine tumors, and breast cancer. According to Touhami et al. [147], only one severe occurrence of bilateral optic neuropathy and posterior reversible encephalopathy syndrome in a patient using everolimus necessitated the prompt withdrawal of the medication.

### 3.16. Janus-Associated Kinase 2 Inhibitors

Janus-associated kinase 2 (JAK2) is a tyrosine kinase that, when mutated, disrupts the JAK-STAT signaling pathway, resulting in the uncontrolled proliferation of erythroid, granulocytic, and megakaryocytic progenitors [148].

Only ruxolitinib and tofacitinib were found to cause retinal side effects. In particular, ruxolitinib has been mainly associated with infectious side effects, specifically bilateral toxoplasmosis retinitis, cytomegalovirus retinitis, and aspergillosis-associated retinal necrosis, requiring drug discontinuation [149,150,151].

The increased risk of severe viral infection has also been observed with tofacitinib in two case reports of cytomegalovirus retinitis, which needed drug discontinuation and systemic therapy with antiviral drugs [152,153].

## 4. Discussion

The development of novel targeted anticancer medications has significantly modified cancer treatment approaches and improved patient outcomes.

Nevertheless, anticancer medications may occasionally cause treatment-related side effects affecting the eyes (Table 1).

Even if new anticancer medications appear to be better tolerated than conventional and traditional chemotherapy (Table 2), posterior segment ocular adverse effects are still possible and how to properly manage them should be known.

Significant cases of uveitis or VKH-like syndrome linked to BRAF inhibitors or immunotherapy, vascular occlusions, or ischemic optic neuropathy caused by MEK inhibitors are of the greatest concern because they can cause permanent deterioration of vision, or even blindness. Drug discontinuation does not need to be permanent in many cases of mild-to-moderate ocular toxicity (uveitis, macular edema); moreover, depending on the circumstances, the patient may also be rechallenged at the same dose or a lower one.

On the other hand, for toxicities that are temporary (like MEKAR) or that may be controlled with local therapy, treatment discontinuation is not necessary. In some cases, more vigilance is required in the presence of risk factors for retinopathy development, especially during the first 3 months of administration.

Concerning the pathophysiological mechanisms underlying the adverse events, it was not possible to identify or hypothesize them for each class of anticancer drugs, such as for EGFR inhibitors, FGFR inhibitors, HER2 inhibitors, and ALK inhibitors.

Conversely, in some cases, the mechanism underlying the adverse effects is intrinsic to the mechanism of action of the drug itself, as in the case of VEGF inhibitors, which increase the risk of thromboembolic events due to their pharmacological action.

For this reason, it is crucial to know the pharmacological characteristics of each anticancer molecule, especially the newer and more recent ones, in order to be able to predict possible adverse effects and promptly manage them.

Finally, determining the frequency and severity of side effects is a key concern in pharmacological risk–benefit analysis. For this reason, finding predictive factors for retinal adverse events is a crucial challenge. This would allow for either closer ophthalmological follow-up when necessary or, if feasible, a lower dosage of an anticancer medication or an alternative anticancer treatment.

To date, the only safe method available to choose patients for a novel anticancer treatment is to perform a thorough ophthalmological examination in the attempt to find any risk factors, as no research has yet focused on the identification of such predictors.

## 5. Conclusions

In conclusion, oncologists and ophthalmologists should be aware of the frequency and features of potential ocular adverse events that may occur in patients receiving anticancer drugs, particularly for newer and modern therapies. In this way, clinicians may ensure appropriate management and minimize potentially crippling retinal and choroidal complications. However, to better understand how retinal side effects affect the clinical course of oncologic patients and, ideally, to find potential predictors for these events, larger case–control studies are necessary.

## Figures and Tables

**Table 1:** Summary of the main ocular posterior segment complications caused by novel anticancer drugs.

Agent	Malignancy	Posterior Segment Adverse Effects	Management
**Epidermal Growth Factor Receptor Inhibitors**			
*Gefitinib* *Brigatinib*	Non-small-cell lung cancer Pancreatic cancer Colorectal cancer Head and neck cancers	Macular edema Floaters Macular edema	No need of drug discontinuation. No need of drug discontinuation.
**Fibroblast Growth Factor Receptor Inhibitors**			
*Erdafitinib*	Urothelial carcinoma	Central serous retinopathy Bilateral exudative retinal detachment and edema Retinal pigment epithelium detachment	Permanent drug discontinuation or dose reduction.
**Human Epidermal Growth Factor 2 Inhibitors**			
*Trastuzumab*	Breast cancer	Bilateral macular ischemia and edema Papilledema Retinal hemorrhage Retinal artery occlusion Retinal vein occlusion	Consider drug discontinuation.
**Immune checkpoint inhibitors**			
*Ipilimumab* *Durvalumab* *Nivolumab* *Pembrolizumab* *Atezolizumab* *Cemiplimab*	Hodgkin lymphoma Colorectal cancer Gastric cancer Urothelial carcinoma Non-small-cell lung cancer Hepatocellular carcinoma Head and neck cancers	Vogt-Koyanagi-Harada (VKH)-like syndrome Panuveitis Posterior uveitis Neurosensory detachment without choroidopathy Multifocal neurosensory detachments with choroidal thickening Choroidal neovascular membranes Immunological retinopathy Optic neuritis	Topical, periocular, or systemic corticosteroids. Intravitreal corticosteroids are also used to treat any related macular edema, in addition to the drug’s discontinuation in severe cases. Intravenous methylprednisolone and high-dose oral prednisone. In some cases, intravenous immunoglobulin and plasma exchange therapy are also necessary, as well as stopping the drug’s administration.
**Mitogen-activated protein kinase inhibitors**			
*Trametinib* *Cobinetimib* *Binimetinib* *Mirdametinib* *Refametinib* *Pimasertib*	Melanoma Colorectal cancer Non-small cell lung cancer Hepatocellular carcinoma Ovarian cancer	Mitogen-activated protein kinase inhibitor-associated retinopathy Retinal vein occlusion posterior uveitis and panuveitis, Vogt–Koyanagi–Harada-like syndrome	No need of drug discontinuation. Dose reduction only in symptomatic cases. Anti-vascular endothelial growth factor injections in case of macular edema and drug discontinuation. Topical, periocular, or systemic corticosteroids, as well as cycloplegic agents. If there is no response, therapy may be withheld for up to 6 weeks. Subsequently, if improvement occurs, therapy is then resumed; otherwise, it is permanently discontinued.
**BRAF Inhibitors**			
*Vemurafenib* *Dabrafenib* *Encorafenib*	Melanoma Non-small cell lung cancer Thyroid cancer Colorectal cancer	Panuveitis Vogt–Koyanagi–Harada-like syndrome Ischemic retinal vasculitis Multifocal choroiditis Retinal vein occlusion	Permanent drug discontinuation.
**Aromatase inhibitors**			
*Anastrozole* *Letrozole* *Exemestane*	Breast cancer	Retinal hemorrhages Hemi-central retinal artery occlusion Macular and optic disk edema Crystalline retinopathy	Temporary drug discontinuation or dose reduction.
**Selective Estrogen Receptor Modulator**			
*Tamoxifen* *Toremifene*	Breast cancer	Crystalline retinopathy Macular edema Macular hole Retinal vein occlusion Optic neuropathy Retinal vein occlusion	Only observation. Temporary drug suspension or discontinuation could be considered in symptomatic cases Vitrectomy Therapy cessation Therapy cessation Therapy cessation
**Bruton** **’** **s tyrosine kinase inhibitors**			
*Ibrutinib*	B-cell malignancies	Branch retinal artery occlusion Macular edema	Therapy cessation Drug discontinuation and administration of intravitreal steroids.
**Breakpoint cluster region–Abelson oncogene inhibitors**			
*Imatinib* *Dasatinib* *Ponatinib*	Chronic myeloid leukemia	Macular edema Optic nerve edema Serous retinal detachment Optic neuritis Ischemic maculopathy Optic neuropathy Papilledema	Oral corticosteroids and cessation of the medication. Dose reduction or treatment cessation. Oral corticosteroids and cessation of the medication. Acetazolamide 500 mg twice daily.
**Vascular endothelial growth factor receptor inhibitors**			
*Pazopanib* *Axitinib* *Bevacizumab* *Cabozantinib*	Renal cancer Sarcoma Non-small cell lung cancer Thyroid cancer	Retinal detachment or tears Vitreous/retinal bleeding Optic nerve disease Retinal artery or vein occlusions Impaired retinal circulation, characterized by multiple cotton-wool spots on fundus examination and significant delays in retinal vessel filling Ischemic optic neuropathy Increased thrombotic events	Drug discontinuation. Drug discontinuation. Drug discontinuation. Drug discontinuation.
**Proteasome inhibitors**			
*Bortezomib*	Multiple myeloma	Optic atrophy	Drug discontinuation.
**Anaplastic lymphoma kinase inhibitors**			
*Crizotinib* *Ceritinib* *Alectinib* *Lorlatinib* *Entrectinib*	Non-small cell lung cancer	Vision disorders Retinal hemorrhages Optic neuropathy Vision disorders	Generally benign, decreased over time, and did not necessitate treatment. Only observation. Drug discontinuation. Generally benign, decreased over time, and did not necessitate treatment.
**Fms-Like Tyrosine Kinase 3 Inhibitors**			
*Sorafenib* *Sunitinib* *Gilteritinib*	Acute myeloid leukemia	Retinal arterial and venous occlusions Retinal tear or detachment Retinal/vitreous bleeding Macular edema Optic neuritis Retinal hemorrhages	Generally, drug discontinuation. Drug discontinuation and systemic methylprednisolone. Only observation.
**Mammalian Target of Rapamycin Inhibitors**			
*Everolimus*	Breast cancer Neuroendocrine tumors Renal cell carcinoma	Optic neuropathy	Drug discontinuation.
**Janus-Associated Kinase 2 Inhibitors**			
*Ruxolitinib* *Tofacitinib*	Myelofibrosis	Bilateral toxoplasmosis retinitis Cytomegalovirus retinitis, Aspergillosis-associated retinal necrosis	Drug discontinuation and appropriate systemic therapy.

**Table 2:** Summary of the main ocular posterior segment adverse effects caused by traditional anticancer drugs.

Agent	Malignancy	Posterior Segment Adverse Effects	Management
*Cisplatin*	Small cell lung cancer Cerebral malignant lymphoma Germinal cell tumors	Retinal ischemia with neovascularization Branch retinal artery occlusion Retinal nerve fiber layer decrease Cone photoreceptors dysfunction Optic neuropathy	Drug discontinuation
*Carboplatin*	Lung cancer Breast cancer Renal cancer Ovarian cancer Head and neck cancers	Maculopathy Optic neuropathy	Drug discontinuation
*Carmustine*	Brain tumors Hodgkin lymphoma Myeloma	Retinal artery occlusion Retinal nerve fiber layer infarct Ischemic optic neuropathy Retinal vasculitis	Drug discontinuation
*Nitrogen mustard derivatives*	Hodgkin lymphoma Non-Hodgkin lymphoma	Necrotizing uveitis Papilledema Retinal hemorrhages	Drug discontinuation
*Fludarabine*	Hematologic malignancies	Optic neuritis	Drug discontinuation
*Cytosine arabinoside*	Hematologic malignancies	Cotton wool spots Capillary nonperfusion Macular edema and neovascularization Ischemic retinopathy	Drug discontinuation
*Methotrexate*	Breast cancer Head and neck cancers Leukemia Lymphoma	Optic neuropathy Cotton wool spots Reversible reduction in rod and cone responses	Drug discontinuation
*Pentostatin*	Lymphoma Chronic lymphocytic leukemia	Retinopathy	Drug discontinuation
*Docetaxel* *Paclitaxel*	Head and neck cancers Breast cancer Prostate cancer Gastric cancer	Taxane-induced maculopathy Optic neuropathy	Drug discontinuation
*Vincristine* *Vinblastine* *Vindesine* *Vinorelbine*	Non-Hodgkin lymphoma Hodgkin’s disease Leukemia Ewing’s sarcoma Lung cancer Breast cancer	Optic neuropathy	Drug discontinuation
*Mitotane*	Adrenal cortical carcinoma	Small retinal hemorrhages Retinal edema Papilledema Pigmentary retinopathy	Drug discontinuation
*Retinoic acid derivatives*	Acute promyelocytic leukemia Breast cancer	Night blindness	Drug discontinuation

## Data Availability

No new data were created or analyzed in this study. Data sharing is not applicable to this article.
